# Supplementary material for: The intersection between migration, HIV, and contraceptive use in Uganda: a cross-sectional population-based study
Source: Reprod Health. 2024 May 17;21:65. doi: 10.1186/s12978-024-01796-z (PMC11100193; doi:10.1186/s12978-024-01796-z)
Supplement: Supplementary file 1 — Supplementary Material 1. [file 12978_2024_1796_MOESM1_ESM.docx]

**Supplemental Table 1.** Factors associated with unsatisfied contraceptive demand among 3,417 sexually active female participants in the RCCS with no intention of getting pregnant in the next year – sensitivity analysis excluding condoms.

|  | **n/N (%)** | **Unadjusted PR**  **[95% CI]** | **p-value** | **Adjusted PR**  **[95% CI]** | **p-value** |
| --- | --- | --- | --- | --- | --- |
| **Migration status** |  |  |  |  |  |
| Resident | 1595/2822 (56.52%) | Ref |  | Ref |  |
| In-migrant | 358/595 (60.17%) | 1.06 [0.99, 1.15] | 0.093 | 1.06 [0.98, 1.16] | 0.158 |
| **HIV serostatus** |  |  |  |  |  |
| HIV-seronegative | 1609/2826 (56.94%) | Ref |  | Ref |  |
| HIV-seropositive | 344/591 (58.21%) | 1.02 [0.95, 1.10] | 0.566 | 1.00 [0.89, 1.12] | 0.992 |
| **Interaction term between migration and HIV-positive serostatus** | 75/115 (65.22%) | 1.15 [1.00, 1.31] | 0.050 | 1.10 [0.92, 1.32] | 0.298 |
| **Age** |  |  |  |  |  |
| 15-24 | 588/910 (64.62%) | Ref |  | Ref |  |
| 25-34 | 700/1462 (47.88%) | 0.74 [0.69, 0.80] | <0.001 | 0.83 [0.77, 0.90] | <0.001 |
| 35-49 | 665/1045 (63.64%) | 0.98 [0.92, 1.05] | 0.652 | 1.10 [1.02, 1.20] | 0.016 |
| **Education** |  |  |  |  |  |
| Some primary | 1141/1991 (57.31%) | Ref |  | Ref |  |
| Post primary | 812/1426 (56.94%) | 0.99 [0.94, 1.05] | 0.832 | 1.00 [0.94, 1.06] | 0.945 |
| **Marital status** |  |  |  |  |  |
| Never married | 360/463 (77.75%) | Ref |  | Ref |  |
| Currently married | 1245/2411 (51.64%) | 0.66 [0.62, 0.71] | <0.001 | 0.67 [0.62, 0.72] | <0.001 |
| Previously married | 348/543 (64.09%) | 0.82 [0.76, 0.89] | <0.001 | 0.79 [0.72, 0.87] | <0.001 |
| **SES** |  |  |  |  |  |
| Lowest | 283/444 (63.74%) | Ref |  | Ref |  |
| Low middle | 590/1016 (58.07%) | 0.91 [0.83, 0.99] | 0.037 | 0.92 [0.84, 1.00] | 0.059 |
| High middle | 577/979 (58.94%) | 0.92 [0.85, 1.00] | 0.079 | 0.94 [0.86, 1.02] | 0.159 |
| Highest | 501/976 (51.33%) | 0.81 [0.73, 0.88] | <0.001 | 0.86 [0.78, 0.95] | 0.002 |
| **Religion** |  |  |  |  |  |
| Other | 13/24 (54.17%) | Ref |  | Ref |  |
| Catholic | 1267/2230 (56.82%) | 1.05 [0.72, 1.51] | 0.800 | 1.13 [0.76, 1.67] | 0.558 |
| Protestant/Pentecost | 417/704 (59.23%) | 1.09 [0.75, 1.59] | 0.639 | 1.17 [0.79, 1.75] | 0.438 |
| Muslim | 256/459 (55.77%) | 1.03 [0.71, 1.50] | 0.879 | 1.11 [0.75, 1.67] | 0.597 |
| **Occupation** |  |  |  |  |  |
| Agriculture/housework | 1137/1997 (56.94%) | Ref |  | Ref |  |
| Non agriculture | 816/1420 (57.46%) | 1.00 [0.95, 1.07] | 0.758 | 0.94 [0.89, 1.00] | 0.070 |
| **Current ART use among women living with HIV** |  |  |  |  |  |
| Currently using ART | 75/213 (35.2%) | Ref |  | Ref |  |
| Not currently using ART | 61/184 (33.2%) | 1.09 [0.90, 1.32] | 0.375 | 1.11 [0.92, 1.34] | 0.278 |

PRR=Prevalence risk ratio; adjRR=adjusted prevalence risk ratio; 95% CI=95% confidence interval; Ref=reference
